# Intra-axial Cortical-Based Tumour Presented as Homonymous Hemianopia in a Young Patient: A Diagnostic Dilemma

**DOI:** 10.7759/cureus.64963

**Published:** 2024-07-19

**Authors:** Luqmanhaqim Aminuddin, Wan-Hazabbah Wan Hitam, Shahidatul-Adha Mohamad, Sanihah Abdul Halim, Nur Asma Sapiai

**Affiliations:** 1 Department of Ophthalmology and Visual Science, School of Medical Sciences, Universiti Sains Malaysia, Kubang Kerian, MYS; 2 Brain and Behaviour Cluster, School of Medical Sciences, Hospital Universiti Sains Malaysia, Kubang Kerian, MYS; 3 Department of Internal Medicine (Neurology) School of Medical Sciences, Universiti Sains Malaysia, Kubang Kerian, MYS; 4 Department of Radiology, School of Medical Sciences, Universiti Sains Malaysia, Kubang Kerian, MYS

**Keywords:** dysembryoplastic neuroepithelial tumour, oligodendroglioma, ganglioglioma, pleomorphic xanthoastrocytoma, intra-axial cortical based tumour

## Abstract

Intra-axial cortical-based tumours are rare tumours affecting children and young adults. These tumours can be classified as either low-grade or high-grade, depending on their aggressiveness and rate of growth. We report a case of homonymous hemianopia secondary to an intra-axial cortical-based tumour in a young patient. A 26-year-old lady presented with bilateral blurring of vision for three weeks associated with a headache. Visual acuity was 6/6 in both eyes. Bilateral optic nerve functions were normal. The Humphrey visual field test showed left-homonymous hemianopia. A CT scan and MRI of the brain revealed an intra-axial cortical-based tumor. Differential diagnoses include pleomorphic xanthoastrocytoma (PXA), ganglioglioma, oligodendroglioma, and dysembryoplastic neuroepithelial tumour (DNET). The patient was treated conservatively and closely monitored through clinic follow-up.

## Introduction

Intra-axial cortical-based tumours are rare intracranial neoplasms. These tumours originate from brain cells, such as glial cells, astrocytes, and oligodendrocytes. Most patients with these tumours present with a history of seizures, as they tend to involve the temporal lobe of the brain. Homonymous hemianopia is an atypical presentation of these tumours [1]. The majority of intra-axial cortical-based tumours are low-grade and have a good prognosis with treatment [2]. Intra-axial cortical-based tumours that commonly affect young adults are pleomorphic xanthoastrocytoma (PXA), ganglioglioma, oligodendroglioma, and dysembryoplastic neuroepithelial tumour (DNET).

## Case presentation

A 26-year-old woman presented with blurred vision in both eyes for three weeks. She described an inability to see the left half of her field of vision. Two months prior, she experienced a throbbing headache with a pain score of 10/10, accompanied by nausea and vomiting, and sought help from a local doctor and the emergency department. She was discharged with medications, and her headache gradually improved with analgesics.

The patient had an unintentional weight loss of 4 kg over the past four months and had a reduced appetite. She did not have any eye pain, redness, swelling, or eye discharge. There was no history of fever, headache, nausea, or vomiting. She did not experience any body or limb weakness and denied any high-risk behaviour related to sexual partners or intravenous drug use.

**Figure 1:**
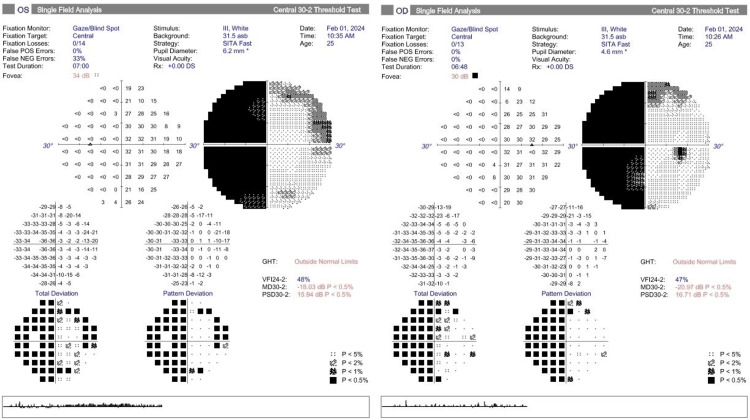
Humphrey visual field test showing homonymous hemianopia.

Clinically, there was the presence of left homonymous hemianopia on the confrontation visual field test. This finding was confirmed by the Humphrey visual field test (Figure 1). Other optic nerve functions were normal, and there was no relative afferent pupillary defect. Both eyes had a best-corrected visual acuity (BCVA) of 6/6. Bilateral extraocular muscle movements were full. The anterior segments of both eyes were normal, and the fundoscopy showed no abnormalities or papilloedema.

**Figure 2:**
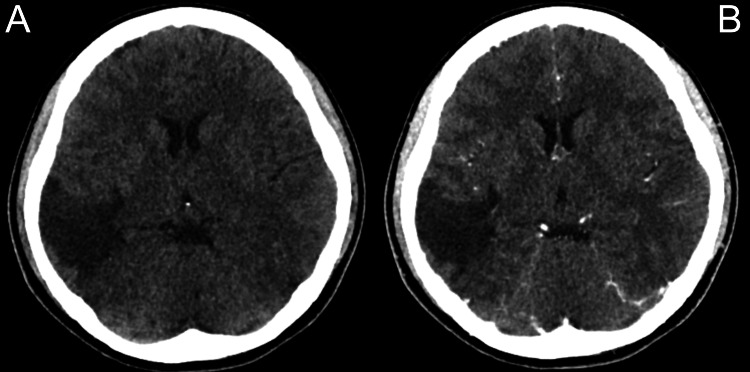
CT scan of the brain, plain (A), and contrasted (B) showing right parieto-temporal lobe encephalomalacia.

**Figure 3:**
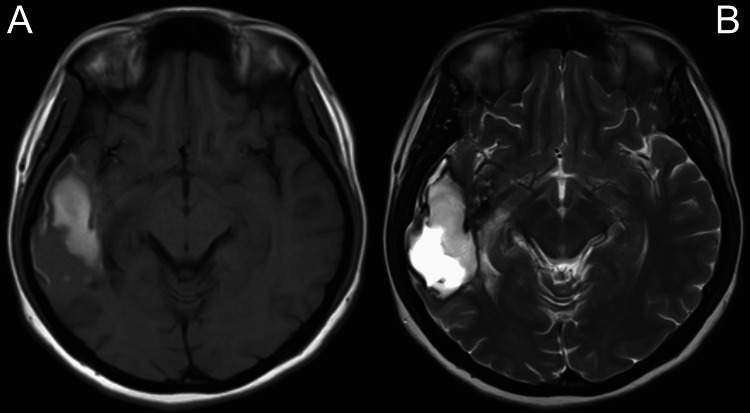
MRI of the brain, T1 sequence (A), and T2 sequence (B) showing right temporal lobe intra-axial cortical base lesions with adjacent mass effect and perilesional oedema.

A contrast-enhanced CT scan of the brain revealed right parieto-temporal encephalomalacia without any midline shift (Figure 2). An MRI showed a solid-cystic intra-axial cortical-based lesion in the right temporal lobe, measuring 5.3 cm × 3.0 cm × 3.4 cm (AP × W × CC), with an adjacent mass effect and perilesional oedema (Figure 3). The differential diagnoses included PXA, ganglioglioma, oligodendroglioma, and DNET. The treatment plan, including the option for a confirmatory brain tissue biopsy, was explained to the patient. However, given the absence of other neurological deficits, she opted not to proceed with the biopsy. She was treated conservatively and scheduled for close follow-up.

## Discussion

Intra-axial cortical-based tumours represent a rare and diverse group of intracranial neoplasms primarily arising from within the brain parenchyma. These tumours often manifest in the temporal lobe, presenting with a variety of neurological symptoms, most commonly seizures, due to their cortical involvement [3]. The presentation of homonymous hemianopia, while atypical, underscores the importance of thorough investigation in young patients exhibiting such visual field deficits.

In our case, the patient was diagnosed with a right temporal lobe intra-axial cortical-based tumour. Among the important intra-axial cortical-based tumours to be considered are PXA, ganglioglioma, oligodendroglioma, and DNET. PXAs are rare and benign brain tumours usually categorised as World Health Organisation (WHO) grade II [4]. PXAs mainly affect individuals in the paediatric and young adult age groups, frequently manifesting as seizures as a result of their typical location in the cerebral cortex. PXAs exhibit a varied histopathological appearance, featuring big, pleomorphic cells that are rich in lipids, hence the name [5]. Surgical resection is the primary treatment modality, in which total removal often leads to long-lasting remission. Nevertheless, it is imperative to have regular follow-ups with imaging, given their position and likelihood of recurring. It is important to note that PXAs often exhibit BRAF V600E mutations, with around 60% of patients containing this specific mutation. The detection of BRAF V600E mutations in PXA has emerged as a key focus for targeted therapy, especially for tumours that recur or cannot be surgically removed [6].

Gangliogliomas are rare brain neoplasms that predominantly occur in children and young adults. Tumours frequently manifest with symptoms such as seizures, as the majority (79%) of tumours tend to develop in the temporal lobe [7]. These tumours are believed to arise from a glioneuronal progenitor and contain both neuronal and glial components [8].

Oligodendrogliomas are gliomas originating from oligodendrocytes, responsible for producing the myelin sheath that insulates nerve fibres in the brain and spinal cord [9]. They are slow-growing tumours predominantly found in adults and are known for their sensitivity to treatment compared to other glioma subtypes [10]. Anaplastic oligodendrogliomas are typically treated with tumour resection, adjuvant radiation, and chemotherapy.

DNETs are benign, rare brain tumours that mostly affect adolescents and young adults and frequently appear with drug-resistant epilepsy. These tumours are most typically situated in the cortical regions of the temporal lobe and are characterised histologically by glioneuronal components and "floating neurons" in a mucinous matrix [11]. The standard therapy is surgical excision, which usually results in excellent seizure control and a good prognosis due to the tumour’s benign nature and low recurrence rate.

Radiologically, the tumours have some similarities and also features that are peculiar to the individual type of tumour. The majority of these tumours show heterogenous enhancement in T2 and T1 post-contrasted MRI. Also, calcifications are common in all except PXAs [12]. Both PXAs and gangliogliomas have cystic appearances with enhanced mural nodules, while oligodendromas are mostly solid. DNETs, on the other hand, have an edge-shaped, multinodular, pseudocystic cortical mass and bubbly appearance [13].

The overall prognosis for intra-axial cortical-based tumours is often favourable. However, in certain instances, a high-grade tumour might result in morbidity and death despite receiving medical intervention. Histological confirmation is crucial in determining the tumour's grade.

Conservative management without histological confirmation may impose a risk on the patient. In our case, all the benefits of histological confirmation and the risks of conservative management have been explained in great detail.

## Conclusions

Intra-axial cortical tumours are among the rare tumours clinically presented with homonymous hemianopia. Neuroimaging, i.e., CT scans and MRIs, are very important tools in making a diagnosis. Differential diagnoses to be considered in the young age group are PXA, ganglioglioma, oligodendroglioma, and DNET. Most of these tumours are considered to be low-grade in nature and have a good prognosis with surgical treatment. However, without histological diagnosis, management can be challenging.
